# Climate micro-mobilities as adaptation practice in the Pacific: the case of Samoa

**DOI:** 10.1098/rstb.2022.0392

**Published:** 2023-11-06

**Authors:** Anita Latai-Niusulu, Masami Tsujita, Andreas Neef

**Affiliations:** ^1^ Department of Social Sciences, National University of Samoa, Apia, PO Box 2279, Samoa; ^2^ Development Studies Programme, Centre for Samoan Studies, National University of Samoa, Apia, PO Box 2279, Samoa; ^3^ Development Studies, School of Social Sciences, The University of Auckland, Auckland 1142, New Zealand

**Keywords:** circular migration, micro-mobilities, climate adaptation, climate migration, Samoan mobility, Pacific islands region

## Abstract

Recent debates on climate mobilities have largely ignored the dynamics of mobility patterns including short-distance and short-duration circular movements to enhance adaptative capacity and resilience of households and individuals, enabling them to remain in place despite facing increasingly severe climatic risks. This paper explores Pacific Islanders' climate-related mobilities with reference to cases from Samoa. It first conceptualizes Samoan mobility, which is rooted in Samoan culture, norms and worldviews, and then uses this as a framework to examine ways in which people shift and diversify their residential locations for climate-associated reasons. The study employs a comparative case study approach using conversational (the Pacific-originated *talanoa*-style) interviews with 40 participants in two villages in Samoa—one urban and the other rural. Findings suggest that shifting spatially and temporarily between two residences (a practice called *fa'a-’āigalua*) occurs not only within the village but across villages. Thereby, villagers reduce the risk of incurring physical harm from climate-related disasters, while minimizing the risk of cultural harm from place detachment. Our study challenges the discourse of ‘vulnerable Pacific Islanders' by demonstrating the adaptability of Samoans to changing socio-ecological and climatic circumstances and their ability to develop a variety of climate resilience strategies, including micro-mobilities and circular migration.

This article is part of the theme issue ‘Climate change adaptation needs a science of culture’.

## 1. Introduction

Pacific Islanders have been exposed to many physical and physiological disturbances associated with tectonic and climate change-related environmental transformations [1,2]. Socio-economic pressures caused by neoliberal development processes have added another layer of challenges, including the promotion of the economic use of customary land by multilateral financial institutions and of greater access for foreign direct investment in tourism and other industries [3–5]. Pacific Islanders’ adaptability, extended kin-based networks and family-oriented social structures that provide access to land, natural resources and remittances have been significant strategies for survival in a multi-risk environment. Being able to move from one place to another, in particular, has been a crucial livelihood strategy in the face of various internal and external pressures, including climate change. Therefore, understanding Pacific Islanders' mobility patterns is imperative to develop policies that help enhance their climate resilience.

This study explores Pacific Islanders’ mobility patterns with reference to two cases from Samoa. It first conceptualizes Samoan mobility, which is rooted in Samoan culture and principles, and then uses it as a framework to examine ways in which people increasingly shift and diversify their residential locations for climate-associated reasons. Ultimately, the study challenges the discourse of vulnerable Pacific Islanders by demonstrating the adaptivity of Samoans to given circumstances and their ability to develop a variety of climate resilience strategies. This study employs a comparative case study approach using the Pacific-originated semi-structured, conversational interview method called *talanoa* with people in two villages in Samoa—one is urban and the other rural. The field data were collected between June and August 2021.

## 2. Conceptualizing Samoan mobility

Mobility and migration have been commonplace across the Pacific for centuries. Family connections between Tonga, Samoa and Fiji and the existence of various diasporic communities in Samoa are evidence of these historic movements [6,7]. The late Tongan–Fijian scholar Epeli Hau'ofa emphasized that prior to colonization, Pacific mobility and movement between a ‘sea of islands’ connected nations and created networks that transcended the colonial western construct that viewed the Pacific islands as miniscule dots in a vast ocean [8]. In a similar vein, the late population geographer Murray Chapman asserted the inapplicability of conventional migration theories that employed dualistic frames of reference like origin–destination or push–pull to understand the complex and kinship-driven movements of Pacific Islanders [9,10]. He called for alternative ways of understanding the circular and fluid nature of Pacific mobility by looking into the foundation of a society in which the system of population movements is deeply rooted [10].

Circular mobility, inherited from the study of wage–labour mobility in south–central Africa, refers to the movement of continual oscillating back and forth between homes and other places for a variety of reasons. However, the basic principle of Pacific Islanders' circular movements is a territorial separation of obligations, activities and resources [11]. Such separation provides geographically expanded opportunities, including wage employment, commerce, medicine, education, religion and politics. At the same time, circulation provides the security associated with ‘home’ through access to land, local resources, housing materials and trading items, through kinship affiliation, and common values and beliefs [11]. In this context, 'home' means one's locus point—whether it is a natal community, tribe, nuclear family or extended family—at which all movements begin and terminate, and from which circulation continues to occur. These movements are fluid and impermanent as the circulation occurs both spontaneously and customarily at multiple locations and multiple scales.

In the case of Samoa, circular movements that occur at multiple scales are often intertwined deeply with multilayered kinship connections that transcend physical boundaries and international borders. Seasonal work schemes also provide opportunities to travel overseas for short periods of time, accelerating transnational movements. Consequently, Samoa has become what Meleisea [12, p. 191] calls a ‘nation without geographic boundaries'. Lilomaiava-Doktor [13] explains the circular, kinship-oriented Samoan movements with the concept of *malaga* [journeying or traveling back and forth]. *Malaga* is the word commonly used as an equivalent to the English term migration. However, it implies the notion of circularity—both visiting and returning locally and internationally, regardless of the length of time. In their circular movements, neither home nor temporary places of residence are static geographic locations [13]. Rather, the meaning of home is flexible as it refers to the place where one's lineage originates and can also refer to one's country, region, village or household, according to the context. Because Samoans in general are firmly rooted in their genealogical place of origin, they do not necessarily perceive of their *malaga* or shifting of their residential locations as living between two places with no static single place to call home [13].

The key role *malaga* plays is to pay respect and maintain social relationships between ‘*āiga* [family or extended family], *matai* [chiefly titleholders] and family land. ‘*Āiga* means family, both nuclear and extended. The extended and semi-subsistent nature of Samoan households allows for diversified ways of earning income and accessing resources that have been crucial aspects to many families, for example, buying freehold land. ‘*Āiga* also is the foundation of the *fa'amatai,* Samoa's chiefly system which is based on Samoan traditions and customary laws. Every ‘*āiga* is headed by a *matai* who governs the use, distribution and protection of the customary land that belongs to ‘*āiga* [14]*.* About 80% of the land in Samoa is customary land, while 12% is freehold land, and about 7% is government land. All Samoans can claim traditional rights to occupy or use customary land through both maternal and paternal lineages. In a typical village setting, customary land can be divided into four categories of residential area, residential backyard, plantation and forest (and lagoon in some coastal villages) [14,15]. All categories are under the protective and collective authority of village, while plantation land can be allocated by a *matai* to his or her ‘*āiga* for cultivation. This organization of land in the village makes it flexible for a family to shift their house location around according to the need, which helps build their resilience against climate change.

To maintain their ties to ‘*āiga, matai* and land, Samoans *malaga:* they move back and forth to strengthen family connections and cultivate social relationships between kin members, irrespective of their geographical locations and for the collective welfare of their family [13,16]. However, such circular movements are being increasingly threatened by neoliberal attempts to reform and financialize customary land arrangements. The previous Samoan government, in partnership with multilateral funding agencies, introduced a customary land lease registry, established mortgage markets for customary land leases and removed the Land and Titles Court from provisions in the Constitution of Samoa for protection of individual rights [4,5]. Studies on Efate Island in Vanuatu—where 80% of coastal land under customary control has been leased by foreign investors over a period of 75 years for tourism facilities and expatriate residential development—provide a cautionary tale for the case of Samoa [17].

## 3. Micro-mobilities and the cultural practice of *Fa'a-'āigalua*

While it explains well the circularity of Samoan mobility, the concept of *malaga* does not fully capture or illuminate micro-mobilities or localized movements that Samoans practice to avoid and adapt to current and future disturbances, including climate change. Moreover, *malaga* does not encapsulate the role of social and natural disturbances in prompting people to shift between residences, within their home villages or across islands. To understand further their micro-mobilities and how these practices enhance the climate change adaptability of Samoans, this study draws on the Samoan cultural practice of *fa'a-‘āigalua*. *Fa'a-‘āigalua* literally means ‘to be with two families' or ‘having two or more residences or families’. It is a cultural practice that enables Samoans to shift back and forth between two houses, families or residential locations, flexibly and spontaneously, not only within the village but also across villages [18]. It is another form of circular migration that occurs mostly at the local scale. Some families may leave their residences for whichever reason and move to other places or move in with relatives living in other villages. Their movements, however, are often temporary. As the term *fa'a-‘āigalua* indicates, for Samoans, living in different residential locations means to be in a continuous state of transitioning between places, with many individuals identifying with several places as ‘home’ rather than one particular place. Sites or previous residences are usually not abandoned, but are often visited even when people no longer reside there [18].

*Fa'a-‘āigalua* is enabled by the system of customary land rights and extended family connections, which allow Samoans to hold two or more parcels of land and diversify their places of residence and plantations [15,18]. For example, many families living on the coastal parts of villages cultivate crops on other parcels of land located inland or even in another village. In this light, *fa'a-‘āigalua* can enhance people's adaptability to changing climate and challenging situations. This study examines how the practice of *fa'a-‘āigalua* works as a climate adaptation strategy.

## 4. The study area

Samoa has 275 villages, of which 192 are considered traditional villages, 48 are sub-villages of traditional villages and 35 are non-traditional villages, which include new settlements and suburban areas [19]. Two research sites—the villages of Salani and Vaitele—were selected to compare perceptions and experiences of mobility between the families who live on customary land in a rural village and those who live on freehold land in a more urban area.

Salani, located on the southeast coast of Upolu, is a traditional coastal village where nearly all families live on customary land and the principles of the traditional and village authority are well maintained. According to the 2016 census, 93% of the village's 88 households live on customary land while 3.4% live on freehold land. Salani is a middle-size village in terms of its population of approximately 550 [20]. Almost all families in the district own plantations producing crops like taro, *ta'amū* (giant taro), banana, coconut and cocoa. The district also has a good marine environment, comprising a wide windward barrier reef, an extensive lagoon with deep blue holes and reef slopes. Most villages in the district have fishery reserves, and fishing is a major source of income for the families that own fishing boats [21].

On 29 September 2009, a magnitude 8.1 earthquake that occurred in the Tongan Trench triggered a massive tsunami, destroying nearly all villages on the southern coast of Upolu including Salani, and took the lives of 149 people. The tsunami also considerably affected the natural landscape of the district. It built up the sand along the mouths of estuaries and blocked the natural flow of the river, which created expanded wetlands and flooding of inland streams. After the tsunami, most families in the district shifted their homes inland from the coast [22]. At the same time, forests continued to be stripped as families moved further inland to cultivate fertile soils for plantations [21].

Vaitele, on the other hand, is an urban settlement located inland on the outskirts of Apia where most of the residents live on freehold land, outside the purview of structured village authority. Vaitele is a fast-growing suburban settlement with a population of over 8000. According to the 2016 census, Vaitele has about 1300 households, 80% of which live on freehold land, while only about 7% live on customary land [20]. Many families living in Vaitele today are descendants of Melanesian labourers who worked on the copra plantations as indentured labour in Vaitele as well as in Mulifanua on the northwest end of Upolu. According to Liki [23], when Samoa's largest copra plantation in Mulifanua was closed in the mid-1980s, 95% of the Melanesian labour community were relocated to Vaitele and given a 50-acre tract of land by the government. Some families of original Vaitele villagers still live in Vaitele, but many are living elsewhere in Samoa [23]. Modern-day Vaitele is a government-planned residential and industrial zone that is home to people moving in from rural areas as well as local and international businesses [15,24]. The industrial complex at Vaitele currently accommodates several local and transnational manufacturing plants including British American Tobacco and Samoa Breweries.

## 5. *Talanoa* methodology

This study employs a comparative qualitative case study approach using *talanoa*-style conversational interviews*. Talanoa* literally means ‘talk freely’ and is a term used across the Pacific Islands. It has been developed into a research methodology suitable to conduct interviews in Pacific Island communities as it replicates a space of everyday life on islands in which people share stories and experiences [25,26]. This method helps remove psychological distance and reduces the power differential between interviewer and interviewee and allowed the participants to share their stories in a relaxed atmosphere.

A total of 40 households were invited to participate in this study. Salani participants were recruited by our research associate who resides in Salani, while one of our researchers approached her church community in Vaitele for their participation. The selection of participants was done on the basis of their willingness and availability to participate, but also on the consideration of gender ratio and age range to mitigate the presence of any research bias. All interviews were conducted in the Samoan language by the research associate. The interviews with the Salani participants were conducted at the participants' respective homes. This provided an ideal *talanoa* setting since the interviews were conducted in a most relaxed way just like a storytelling with their neighbour. All the interviews with the Vaitele participants, except one, were conducted at a hall located within the church compound. Although it was a less ideal *talanoa* setting than the one in Salani, the use of the familiar church hall provided some privacy for the participants to share their stories confidentially and in a relaxed atmosphere.

Two sets of guiding questions were used to allow the participants to share control over the research process. The first set was for the participants who had moved in the past 10–30 years and the other set was presented to those who had never moved. Both sets were drawn from the study's key questions: (i) to what extent can mobility be attributed to climate change; (ii) who is most able to move; and (iii) the benefits and challenges of movements. Interview data were digitally recorded and translated from Samoan to English by the research associate and cross-checked by one of the researchers to avoid nuances getting lost in translation. Translated interview data were analysed separately by two researchers, and then compared and combined into a joint analysis to enhance the validity.

## 6. Findings

### (a) Climate responses and other mobility drivers

The study findings confirmed that it is difficult to isolate climate change as the sole driving factor for movement. The majority of participants from both sites identified multiple reasons for movement interacting with environmental, social, and economic aspects of their daily space. At Salani, only four out of 20 participants attributed their movements entirely to climate change. Higher storm surges and heavier spells of rainfall during cyclones were the main dimensions that caused them to move away from the coastal area to the inland parts of their village. All of them talked about changes in the behaviour of the sea and how the sea has come much closer to their homes compared with when they were young. The sea now flows past some of the coconut and *fetau* (Alexandrian Laurel) trees that were once part of the village coastline. During Cyclone Evan in 2013, seawater surged past the coastline and flowed all the way up and into the participants' outdoor kitchens, their houses and the graves of their ancestors. Additional six participants mentioned various dimensions of climate change such as heavier rainfall and storm surges that were both higher and stronger, especially during cyclones Ofa (in 1990) and Evan (in 2013), as triggers for their movements. But these six participants also stated other, non-climate-related factors, namely the 2009 tsunami and social challenges, as reasons to move.

The six participants who attributed their movements to the 2009 tsunami reported how this disaster destroyed the coastal part of the village and caused them to immediately shift inland, where they cultivated food crops. They, however, also mentioned that they were facing difficulties living in the coastal area due to climate-related events and lack of space due to the growth of family members. One participant said:

It was too crowded and we could not do much with our land as our neighbours' houses and our school building were very close to ours. We lived on a small block. The land was too small and there was not much space to cultivate food crops. The place is also prone to flooding so when it rains, our front yard is filled with water. Our land started flooding when they excavated and raised the area where the school buildings are now located. Our land became low so when it rains there is flooding in front of our house. During recent cyclones and periods of heavy rainfall, we left and lived at the school.

Altogether, at Salani, ten participants fully or partially attributed their movement to climate-associated reasons, while six attributed their movement to the 2009 tsunami. Three identified social challenges relating to family as the main motivation behind their movement. One family has not moved but chosen to remain on the coast. The reason provided for staying put was that the participant's parent used to live in this place and it was conveniently close to the church. While the participant felt that this place was safe for the time being, he also mentioned that they might need to move within the next 20 years due to sea-level rise and coastal erosion.

Similar to the case of Salani, the study found it difficult to isolate climate change as a sole driver of movement among Vaitele participants. However, six out of 20 households attributed their movement either fully or partially to climate-associated reasons. One participant mentioned living near the coast and observing sea-level rise as the reason to move. Two stated a change in the behaviour of the river, and their fear of increasing flooding and the 2009 tsunami triggered their decision to move. Two more participants moved after family homes were destroyed by cyclones. Another participant moved when flooding became more frequent and severe.

The father of one participant had operated his family business at the previous location for the past 30 years. He had a strong attachment to the previous place and was reluctant to move. However, the flooding from a nearby river became more frequent and inundated the ground floor of their house, which was located on lower land.

My father almost lost his life during the 2009 flooding before [Cyclone] Evan. If it had been at night time, my father would have died already, and we didn't have time to escape. We ended up on the roof, [this] was the first flooding where logs ended up in our yard. This was when I realized that this issue is becoming serious and getting worse. Every year, flooding is becoming worse and worse … We moved because of this … . My family found it hard to leave, especially my father, a hardworking man, the land at Lelata has so much sentimental value [for him]. It was more personal to my parents.

Eventually, this family sold a portion of their land to the government and with that money, bought a larger piece of land in Vaitele to which they shifted their family business. The participant mentioned that if the government had taken some actions to alleviate such a disastrous situation, they would not have considered moving.

At Vaitele, seven participants identified social issues, particularly tensions among extended family members, marriage and death, as the reason why they moved from the previous place. For another seven participants, gaining access to freehold land led to their decision to move. Six of them purchased their freehold land and another one was appointed to be a guardian of their family's freehold land. Among these 14 participants, eight participants cited climate-associated environmental changes, including increased intensity and frequency of flooding, strong runoff and land erosion in their previous location. Accordingly, the study found in both sites that although climate change or other hazardous events triggered the decision to move, most participants had various, often intertwined reasons, for changing their residential locations.

### (b) ‘Āiga and micro-moblities

As discussed above, Samoan mobility is rooted in ‘*āiga*, at which all movements begin and terminate and from which circulation continues to occur. This study confirmed that ‘*āiga* is one of the common reasons for shifting residential locations. One participant at Salani said that the passing of their parents made them uncertain about their connections to the places, so they moved. Two participants at Vaitele cited the death of a father and the death of a husband, respectively, as the reasons for changing their residential locations. Three participants at Salani and four at Vaitele mentioned that tensions between extended family members triggered their movements. In the case of Salani, the family size had increased due to marriage and having children, causing a lack of space to accommodate the needs of all family members and creating tensions, at times, between family members living together. These participants commented on the importance of married couples having their own space, whether it be a small open house on the same land or moving to a new place, to develop their own residence. One participant said:

We wanted to develop our own family. My husband wanted us to move away so we can be independent and work on our own family development. Living together with other married couples under one roof is problematic and challenging in our experience so it was best to move.

Similar experiences with family tensions were shared by those participants whose triggers to move were climate change and the 2009 tsunami. One female participant at Vaitele, who was a *fafine nofotane* (a woman who married into her husband's family), lived together with a big extended family on their crowded customary land. She felt her physical and psychological space was confined as she had no time to rest and she felt that her husband's family always interfered with what she and her husband wanted to do. At that time, she observed changes in the behaviour of the nearby river and experienced increased flooding there at her husband's place. Ultimately, the 2009 tsunami triggered their decision to move for good. They moved to Vaitele, where they purchased the land in 2010. She now feels free from traditional discrimination against *nofotane* and released from fear of the floods that they experienced in the previous place. A male participant lived with his own extended family but experienced similar tensions among family members living together. He described the move to the current location as *lagona o le saoloto* (feeling a sense of freedom), referring to it being a safer place in terms of flooding and with no one to interfere with his own family decisions.

For these participants, environmental change was a trigger for movement but escaping from extended family living was an additional factor that made them content with their decision. Yet, at the same time, several participants mentioned valuable assistance provided by their family and relatives, which enabled them to move. Although it causes tensions, one of the strengths of an extended family household is its diversified livelihoods. At both sites, the majority of households depend on multiple sources of income. These include the salary of family members working in public or private sectors, irregular income from casual work and selling crops or food, and remittances from members working overseas. Many of the surveyed households have a member who is currently working in New Zealand or Australia under these countries' seasonal work schemes. These family members contribute greatly to the development of the family and their movements. This confirmed that Samoan mobility is, either constructively or unproductively, truly driven by ‘*āiga*.

### (c) Micro-mobility patterns

**Figure 1.**
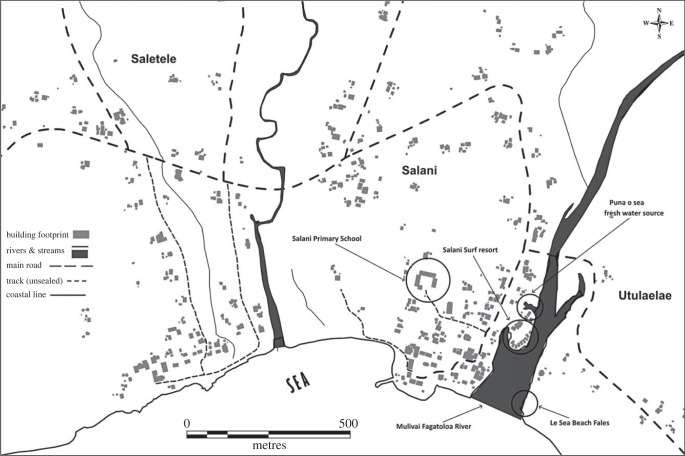
Map depicting the spread of houses at Salani village.

Figure 1 shows the expansive building footprint of Salani village, with houses concentrated at the river mouth and along main roads and unsealed tracks further inland. This pattern reflects the micro-mobility and multilocality of households.

At Salani, 17 out of the 20 households located close to the sea and rivers have shifted their residential locations to the inland parts of the village. Two households moved from the urban area to reside in the inland parts of Salani, and one household moved from the eastern to the western side of the Salani river. Most participants traced the beginnings of their movements to the occurrence of environmental challenges such as the cyclones of the 1990s and the 2009 tsunami, which destroyed their homes and other properties. One participant explained how he felt when the tsunami destroyed the coastal part of Salani.

I was at the plantation when the tsunami hit. As I walked towards the coastal part of the village, I saw great destruction. There was nothing left of our home … Now I thank God we have moved inland. It was better to leave the coast as the tsunami took everything.

Their movements inland have been comparatively quick and easy because they moved within a short distance, from coastal to inland areas, and they did not pay any money for these lands. Only a few families mentioned asking for permission, from their *matai* or other family members, to live in the places where they currently reside. Others shifted their houses to where they used to cultivate their crops. Such short-distance movement, endorsed by the customary nature of land ownership, has enhanced the adaptive capacity and resilience of Salani residents.

Unlike the Salani case in which nearly all the participants shifted their residential locations within the same village or district, the movement pattern of Vaitele participants varied. They moved to Vaitele from different parts of Samoa within the past 50 years. The study found no specific pattern of movement in relation to age, gender, and year of moving. Having access to land is the common enabler of mobility for nearly all Vaitele participants. In other words, they moved to the current location because they had access to a piece of land or had a family who lives there. One participant, for example, had his house built on family-owned freehold land where flooding was frequent. Access to his own freehold land in Vaitele triggered his decision to move, while climate change also played a role in his decision. Nine of the participating households are descendants of Melanesian labourers who had access to purchase land in Vaitele at an affordable price. The case of Vaitele confirmed the importance of having access to own land—freehold land in particular—in order to move and mitigate climate risks. This, however, does not necessarily support the argument that landless urban Samoans are at the highest risk of climate change, because, as discussed in the following section, the practice of *fa'a-‘āigalua* provides geographically expanded opportunities.

## 7. Discussion: *fa'a-‘aigalua* as climate adaptation strategy

The study explored the ways in which *fa'a-‘āigalua* enhanced the resilience of the surveyed households to current and future climate-related challenges. In the case of Salani, families were able to shift their residence quickly from the coastal area to the inland parts of the village not only because of their customary right to access inland parcels, but also because the practice of *fa'a-‘āigalua* made it easy for them to shift their residential location gradually. Many of those who shifted their residence spoke about the difficulties of cutting down trees, clearing bushes, flattening the land and connecting water and electricity in order to make the new place livable. While these processes take time and money, *fa'a-‘āigalua* enabled them to move back and forth between the previous and new locations while preparing the new house. Gradually shifting their residence helped reduce the family's physical and financial burdens. Even after the new house was completed, they continued to visit the old house located on the coast for various occasions and reasons, including to attend church there or to go fishing. As demonstrated by the testimony of the Salani participants quoted earlier, their short-distance and circular movements underpinned by *fa'a-‘āigalua* secured their personal safety and mental wellbeing from experiencing more disasters such as storm surges and flooding during cyclones. In addition, their movements had helped them to avoid future family conflicts and enabled them to access spacious lands to build their homes and cultivate food crops for their families now and in future.

The Vaitele participants shared similar experiences of moving back and forth between two residential locations while they were clearing uncultivated land and settling down at the new place. This gradual and partial shifting made their moving easier both physically and financially as it provided them with flexible options. One participant testified that:

[It] was good transitioning for us. We didn't all come at once. My older sister stayed back [at the previous place] and continued our plantation and so was able to supply us … My children now returned to Aleisa [previous place] and work on the plantation on a 50-acre land to financially support us.

The stories of other participants, on the other hand, illustrate how *fa'a-‘āigalua* provides a temporary shelter for those who are left behind at vulnerable places. One participant said:

I look with sympathy to those still living at Vaimoso [her previous place of residence]. Children lift up furniture when the water floods. We bring our relatives here when the water floods until it goes down, then we take them back. During the tsunami, we also went there to bring our in-laws and relatives here.

Another participant said:

My older brother still lives there [previous place] but when it is close to the rainy season they come here [laughs] then afterwards they move back. He has become used to do that. I love to have him here but he doesn't want to leave there.

Accordingly, *fa'a-‘āigalua* provided the geographically expanded choices of their residential locations and enabled Samoans, either living in or outside of traditional villages, to remain in place despite facing increasingly severe climate risks. In short, the benefits of *fa'a-‘āigalua* outweigh some of the challenges faced by the participants.

As discussed by Pisor [27] and Waring [28], if cultural adaptation means a solution that emerges from the community and is embedded in the everyday living space, identifying cultural adaptation of a society is crucial to weather the effects of climate-associated changes [2]. For Samoans, endorsed by the customary nature of their land system and extended family networks, *fa'a-‘āigalua* has been a cultural adaptation strategy in response to environmental changes and social transformations.

## 8. Conclusion

The current literature on Pacific Islanders' circular migration focuses mostly on inter-island and international movements between islands and neighbouring countries, such as New Zealand, Australia and the USA [29–31]. Much of such literature and discussion on climate adaptation assert that these macro-scale movements should be strategies and long-term solutions for climate change in the Pacific Islands. However, findings from this study demonstrate that Pacific Islanders, particularly those inhabiting tropical high islands like Samoa, have long-practised short-distance, circular migration as an adaptive strategy to avoid and reduce risks posed by environmental challenges including climate-associated changes. Micro-mobilities have been a crucial livelihood strategy of Pacific Islanders for decades, even before climate change became a global and local concern. Given the flexibility and sustainability of their long-practised cultural adaptation based on micro and circular movements, Pacific Islanders are less vulnerable to climate change and less in need of relocation than emphasized in expert discussions and popular discourse.

Samoans’ micro-scale, circular movements like *fa'a-’āigalua,* enabled by customary land ownership systems and supported by family-oriented cultural practices, are simple, affordable and sustainable while providing geographically expanded opportunities for living in at-risk environments. As demonstrated in the study findings, retaining customary land ownership is important as it ensures the continuation of these flexible and spontaneous movements in response to environmental challenges. Hence, both international organizations and local governments should refrain from policies that pose a threat to customary land rights. Rather, they should develop support systems whereby even the most vulnerable members of ‘*āiga* are able to access parcels of land where they can shift their residence and develop multi-local livelihood strategies when future challenges arise.

## Data Availability

Data are available from the Dryad Digital Repository: https://doi.org/10.5061/dryad.k98sf7mcv [32]. This is part of the Climate Change Adaptation Needs a Science of Culture data portal on the Dryad Digital Repository: https://doi.org/10.5061/dryad.bnzs7h4h4 [33]. Data collected for our study are relevant to the study of climate change adaptation as our study aims to explore mobility-related climate adaptation strategies practiced by Pacific Islanders.

## References

[1] Lough J, Gupta AS, Power SB, Grose MR, McGree S. 2015 Observed and projected changes in surface climate of tropical Pacific Islands. In Vulnerability of Pacific agriculture and forestry to climate change (eds M Taylor, A McGregor, B Dawson), pp. 47-101. Noumea, New Caledonia: Secretariat of the Pacific Community.

[2] Neef A, Benge L, Boruff B, Pauli N, Weber E, Varea R. 2018 Climate adaptation strategies in Fiji: the role of social norms and cultural values. World Dev. **107**, 125-137. (10.1016/j.worlddev.2018.02.029)

[3] Ravuvu A, Thornton A. 2016 Beyond aid distribution: aid effectiveness, neoliberal and neostructural reforms in Pacific Island countries. In Assessing the impact of foreign aid: value for money and aid for trade (eds V Jakupec, M Kelly), pp. 79-93. Cambridge, MA: Academic Press.

[4] Meleisea M, Schoeffel P. 2022 Sāmoan custom, individual rights, and the three 2020 acts: Reorganizing the Land and Titles Court. J. Pac. Hist. **57**, 439-450. (10.1080/00223344.2022.2058475)

[5] Raymond EL. 2023 The role of the diaspora: Malaga, vā, and contesting the financialization of customary land in Samoa. Urban Geogr. **44**, 295-297. (10.1080/02723638.2022.2128580)

[6] Davidson JW. 1969 Settlement patterns in Samoa before 1840. J. Polyn. Soc. **78**, 44-82.

[7] Tapu-Qiliho FB. 2017 Tuvaluan diaspora within Oceania: ethnic identity and belongingness in the margins. Thesis, Doctor of Philosophy, University of Otago, Dunedin, New Zealand.

[8] Hau'ofa E. 1994 Our sea of islands. Contemp. Pac. **6**, 148-161.

[9] Chapman M. 1991 Pacific Island movement and socioeconomic change: Metaphors of misunderstanding. Popul. Dev. Rev. **17**, 263-292. (10.2307/1973731)

[10] Chapman M. 1995 Island autobiographies of movement: alternative ways of knowing? Population series, no. 315. Honolulu, HI: East-West Center.

[11] Chapman M, Prothero RM. 1985 Circulation between ‘home’ and other places: some propositions. In Circulation in population movement: substance and concepts from the Melanesian case (eds M Chapman, RM Prothero), pp. 1-12. London, UK: Routledge.

[12] Meleisea M. 2000 Governance, development and leadership in Polynesia: a microstudy from Samoa. In Governance in Samoa (eds E Huffer, A So'o), pp. 189-200. Canberra, Australia: Asia Pacific Press.

[13] Lilomaiava-Doktor S. 2009 Beyond ‘migration’: Samoan population movement (*malaga*) and the geography of social space (*vā*). Contemp. Pac. **21**, 1-32. (10.1353/cp.0.0035)

[14] Taua'a S. 2014 The Samoan fa'amatai system: social protection and governance issues. J. Pac. Stud. **34**, 59-76.

[15] Taua'a S, Schoeffel P. 2019 Town as village: urbanisation, governance, and neotraditionalism in Samoa. J. Samoan Stud. **9**, 7-20.

[16] Lilomaiava-Doktor S. 2015 Journeyings: Samoan understanding of movement. In Oceanian journeys and sojourns (ed. JA Bennett), pp. 67-92. Dunedin, New Zealand: Otago University Press.

[17] Neef A. 2021 The contentious role of tourism in disaster response and recovery in Vanuatu. Front. Earth Sci. **9**, 771345. (10.3389/feart.2021.771345)

[18] Latai-Niusulu A. 2017 Exploring resilience to climate change and other environmental challenges in Samoan communities. Thesis Doctor of Philosophy, Dunedin, New Zealand: University of Otago.

[19] Fiti-Sinclair R, Meleisea M, Schoeffel P. 2017 Women and political participation: The 2016 election in Samoa. Apia, Samoa: National University of Samoa.

[20] Samoa Bureau of Statistics (SBS). 2016 Population and housing census. Apia, Samoa: SBS.

[21] Ministry of Natural Resources and Environment (MNRE). 2018 Community integrated management plan implementation guidelines: Falealili East – Upolu. Apia, Samoa: MNRE.

[22] Hennings W. 2017 The reconstruction of a Samoan village: Quest for the spatial narration of the mythological origin and the social structure of Poutasi. J. Samoan Stud. **7**, 38-62.

[23] Liki A. 2015 Women as kin: Working lives, living work and mobility among Samoan teine uli. In Oceanian journeys and sojourns (ed. JA Bennett), pp. 126-159. Dunedin, New Zealand: Otago University Press.

[24] Sansom G. 2013 Principles for effective local government legislation: lessons from the commonwealth pacific. London, UK: Commonwealth Secretariat.

[25] Vaioleti TM. 2006 Talanoa research methodology: a developing position on Pacific research. Waikato J. Educ. **1**, 21-34.

[26] Suaali'i-Sauni TM. 2012 To talanoa, fa'afaletui, or not? Reflections on the development of Pacific research methods. Va'aomanu Pasifika Units VASA seminar series. Wellington, New Zealand: Victoria University of Wellington.

[27] Pisor A, Lansing J, Magargal K. 2023 Climate change adaptation needs a science of culture. Phil. Trans. R. Soc. B **378**, 20220390. (10.1098/rstb.2022.0390) PMC10505856 37718608

[28] Waring T, Niles M, Kling M, Miller S, Hébert-Dufresne L, Sabzian H, Gotelli N, McGill BJ. 2023 Operationalizing cultural adaptation to climate change: contemporary examples from United States agriculture. Phil. Trans. R. Soc. B **378**, 20220397. (10.1098/rstb.2022.0397) PMC10505858 37718600

[29] Oakes R. 2019 Culture, climate change and mobility decisions in Pacific Small Island Developing States. Popul. Environ. **40**, 480-503. (10.1007/s11111-019-00321-w)

[30] Dun O, McMichael C, McNamara K, Farbotko C. 2022 Investing in home: Development outcomes and climate change adaptation for seasonal workers living between Solomon Islands and Australia. Migr. Dev. **11**, 852-875. (10.1080/21632324.2020.1837535)

[31] Yates O, Groot S, Manuela S, Neef A. 2023 ‘There's so much more to that sinking island!’: Restorying migration from Tuvalu and Kiribati to Aotearoa New Zealand. J. Community Psychol. **51**, 924-944. (10.1002/jcop.22928) 36004412

[32] Latai-Niusulu A, Tsujita M, Neef A. 2023 Data from: Climate micro-mobilities as adaptation practice in the Pacific: the case of Samoa. Dryad Digital Repository. (10.5061/dryad.k98sf7mcv) PMC10505846 37718607

[33] Latai-Niusulu A, Tsujita M, Neef A. 2023 Data from: Climate micro-mobilities as adaptation practice in the Pacific: the case of Samoa. Dryad Digital Repository. (10.5061/dryad.bnzs7h4h4) PMC10505846 37718607

